# 2-Chloro-*N*′-(2-hydr­oxy-4-methoxy­benzyl­idene)benzohydrazide

**DOI:** 10.1107/S1600536809009659

**Published:** 2009-03-19

**Authors:** Qianfeng Weng, Lei Zhao

**Affiliations:** a College of Chemistry and Chemical Engineering, Liaoning Normal University, Dalian 116029, People’s Republic of China

## Abstract

In the title compound, C_15_H_13_ClN_2_O_3_, the dihedral angle between the two benzene rings is 82.09 (10)° and an intra­molecular O—H⋯N hydrogen bond occurs. In the crystal structure, N—H⋯O hydrogen bonds link mol­ecules into chains propagating in [100].

## Related literature

For related structures, see: Fun *et al.* (2008 ▶); Ali *et al.* (2007 ▶); Zhi & Yang (2007 ▶).
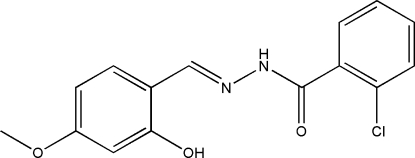


## Experimental

### 

#### Crystal data

C_15_H_13_ClN_2_O_3_
*M* *_r_* = 304.72
Triclinic, 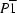

*a* = 5.002 (1) Å
*b* = 10.866 (2) Å
*c* = 13.169 (3) Å
α = 83.946 (3)°
β = 81.721 (4)°
γ = 89.540 (3)°
*V* = 704.3 (2) Å^3^
*Z* = 2
Mo *K*α radiation
μ = 0.28 mm^−1^
*T* = 298 K
0.13 × 0.12 × 0.10 mm

#### Data collection

Bruker SMART 1000 CCD diffractometer
Absorption correction: multi-scan (*SADABS*; Bruker, 2001 ▶) *T*_min_ = 0.964, *T*_max_ = 0.972
4214 measured reflections
3017 independent reflections
2342 reflections with *I* > 2σ(*I*)
*R*_int_ = 0.012

#### Refinement

*R*[*F* ^2^ > 2σ(*F* ^2^)] = 0.043
*wR*(*F* ^2^) = 0.117
*S* = 1.03
3017 reflections
195 parameters
1 restraint
H atoms treated by a mixture of independent and constrained refinement
Δρ_max_ = 0.19 e Å^−3^
Δρ_min_ = −0.28 e Å^−3^

### 

Data collection: *SMART* (Bruker, 2007 ▶); cell refinement: *SAINT* (Bruker, 2007 ▶); data reduction: *SAINT*; program(s) used to solve structure: *SHELXTL* (Sheldrick, 2008 ▶); program(s) used to refine structure: *SHELXTL*; molecular graphics: *SHELXTL*; software used to prepare material for publication: *SHELXTL*.

## Supplementary Material

Crystal structure: contains datablocks global, I. DOI: 10.1107/S1600536809009659/hb2929sup1.cif

Structure factors: contains datablocks I. DOI: 10.1107/S1600536809009659/hb2929Isup2.hkl

Additional supplementary materials:  crystallographic information; 3D view; checkCIF report

## Figures and Tables

**Table 1:** Hydrogen-bond geometry (Å, °)

*D*—H⋯*A*	*D*—H	H⋯*A*	*D*⋯*A*	*D*—H⋯*A*
O2—H2⋯N2	0.82	1.86	2.583 (2)	146
N1—H1⋯O1^i^	0.897 (10)	1.976 (14)	2.817 (2)	156 (2)

Symmetry code: (i) 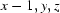.

**Fig. 1.**
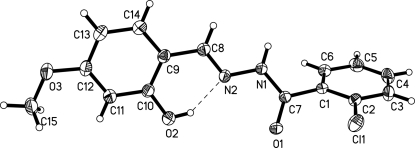
The molecular structure of (I) showing 30% probability displacement ellipsoids for the non-hydrogen atoms. The H bond is shown as a dashed line.

## supplementary crystallographic information

### Comment

Recently, the crystal structures of hydrazone compounds have been widely studied (Fun *et al.*, 2008; Ali *et al.*, 2007; Zhi & Yang, 2007). In this paper, the structure of the title compound, (I), is reported.

In (I), Fig. 1, the dihedral angle between the two benzene rings is 97.9 (2)°. There is an intramolecular O–H···N hydrogen bond (Table 1) in the molecule.

### Experimental

The compound was prepared by the reaction of equimolar quantities (1.0 mmol each) of 2-hydroxy-4-methoxybenzaldehyde and 2-chlorobenzohydrazide in methanol (100 ml) for 2 h at room temperature. The solution was kept in air for two weeks, forming yellow blocks of (I).

### Refinement

The N-bound H atom was located in a difference Fourier map and was refined with an N–H distance restraint of 0.90 (1) Å. Other H atoms were placed in calculated positions (C–H = 0.93–0.96 Å, O–H = 0.82 Å) and refined using a riding model with *U*_iso_(H) = 1.2*U*_eq_(C) and 1.5*U*_eq_(O2 and C15).

### Crystal data

**Table:**

C_15_H_13_ClN_2_O_3_	*Z* = 2
*M**_r_* = 304.72	*F*(000) = 316
Triclinic, *P*1	*D*_x_ = 1.437 Mg m^−^^3^
Hall symbol: -P 1	Mo *K*α radiation, λ = 0.71073 Å
*a* = 5.002 (1) Å	Cell parameters from 1381 reflections
*b* = 10.866 (2) Å	θ = 2.3–26.1°
*c* = 13.169 (3) Å	µ = 0.28 mm^−^^1^
α = 83.946 (3)°	*T* = 298 K
β = 81.721 (4)°	Block, yellow
γ = 89.540 (3)°	0.13 × 0.12 × 0.10 mm
*V* = 704.3 (2) Å^3^	

### Data collection

**Table:**

Bruker SMART 1000 CCD diffractometer	3017 independent reflections
Radiation source: fine-focus sealed tube	2342 reflections with *I* > 2σ(*I*)
graphite	*R*_int_ = 0.012
ω scans	θ_max_ = 27.0°, θ_min_ = 1.6°
Absorption correction: multi-scan (*SADABS*; Bruker, 2001)	*h* = −6→6
*T*_min_ = 0.964, *T*_max_ = 0.972	*k* = −13→13
4214 measured reflections	*l* = −12→16

### Refinement

**Table:**

Refinement on *F*^2^	Primary atom site location: structure-invariant direct methods
Least-squares matrix: full	Secondary atom site location: difference Fourier map
*R*[*F*^2^ > 2σ(*F*^2^)] = 0.043	Hydrogen site location: inferred from neighbouring sites
*wR*(*F*^2^) = 0.117	H atoms treated by a mixture of independent and constrained refinement
*S* = 1.03	*w* = 1/[σ^2^(*F*_o_^2^) + (0.0529*P*)^2^ + 0.2179*P*] where *P* = (*F*_o_^2^ + 2*F*_c_^2^)/3
3017 reflections	(Δ/σ)_max_ < 0.001
195 parameters	Δρ_max_ = 0.19 e Å^−^^3^
1 restraint	Δρ_min_ = −0.28 e Å^−^^3^

### Special details

**Table:**

Geometry. All e.s.d.'s (except the e.s.d. in the dihedral angle between two l.s. planes) are estimated using the full covariance matrix. The cell e.s.d.'s are taken into account individually in the estimation of e.s.d.'s in distances, angles and torsion angles; correlations between e.s.d.'s in cell parameters are only used when they are defined by crystal symmetry. An approximate (isotropic) treatment of cell e.s.d.'s is used for estimating e.s.d.'s involving l.s. planes.
Refinement. Refinement of *F*^2^ against ALL reflections. The weighted *R*-factor *wR* and goodness of fit *S* are based on *F*^2^, conventional *R*-factors *R* are based on *F*, with *F* set to zero for negative *F*^2^. The threshold expression of *F*^2^ > σ(*F*^2^) is used only for calculating *R*-factors(gt) *etc*. and is not relevant to the choice of reflections for refinement. *R*-factors based on *F*^2^ are statistically about twice as large as those based on *F*, and *R*- factors based on ALL data will be even larger.

### Fractional atomic coordinates and isotropic or equivalent isotropic displacement parameters (Å^2^)

**Table:**

	*x*	*y*	*z*	*U*_iso_*/*U*_eq_	
Cl1	0.46805 (11)	0.16315 (5)	0.36552 (4)	0.05502 (19)	
N1	−0.0337 (3)	0.45450 (15)	0.28647 (12)	0.0395 (4)	
N2	0.0359 (3)	0.54868 (14)	0.20805 (12)	0.0398 (4)	
O1	0.3975 (3)	0.44360 (13)	0.31753 (12)	0.0524 (4)	
O2	0.3019 (3)	0.74949 (15)	0.13643 (11)	0.0581 (4)	
H2	0.2626	0.6867	0.1759	0.087*	
O3	0.1119 (3)	0.94970 (14)	−0.18396 (11)	0.0549 (4)	
C1	0.0671 (4)	0.32016 (16)	0.43192 (14)	0.0348 (4)	
C2	0.2007 (4)	0.20956 (17)	0.45277 (15)	0.0396 (4)	
C3	0.1198 (5)	0.13155 (19)	0.54152 (17)	0.0521 (5)	
H3	0.2105	0.0577	0.5543	0.063*	
C4	−0.0949 (5)	0.1634 (2)	0.61080 (17)	0.0577 (6)	
H4	−0.1477	0.1115	0.6710	0.069*	
C5	−0.2326 (4)	0.2716 (2)	0.59178 (16)	0.0527 (5)	
H5	−0.3786	0.2925	0.6388	0.063*	
C6	−0.1531 (4)	0.34920 (18)	0.50246 (15)	0.0422 (4)	
H6	−0.2480	0.4217	0.4894	0.051*	
C7	0.1614 (3)	0.41011 (16)	0.34009 (14)	0.0357 (4)	
C8	−0.1114 (4)	0.56612 (18)	0.13669 (15)	0.0415 (4)	
H8	−0.2606	0.5153	0.1375	0.050*	
C9	−0.0462 (4)	0.66529 (17)	0.05418 (14)	0.0383 (4)	
C10	0.1562 (4)	0.75315 (18)	0.05729 (14)	0.0400 (4)	
C11	0.2109 (4)	0.84952 (19)	−0.02082 (15)	0.0457 (5)	
H11	0.3426	0.9082	−0.0171	0.055*	
C12	0.0700 (4)	0.85834 (18)	−0.10403 (14)	0.0425 (5)	
C13	−0.1288 (4)	0.7717 (2)	−0.10967 (16)	0.0488 (5)	
H13	−0.2226	0.7771	−0.1660	0.059*	
C14	−0.1851 (4)	0.67777 (19)	−0.03091 (15)	0.0460 (5)	
H14	−0.3202	0.6207	−0.0345	0.055*	
C15	0.3106 (5)	1.0415 (2)	−0.17935 (18)	0.0566 (6)	
H15A	0.2643	1.0815	−0.1176	0.085*	
H15B	0.3173	1.1018	−0.2385	0.085*	
H15C	0.4840	1.0031	−0.1789	0.085*	
H1	−0.205 (3)	0.427 (2)	0.300 (2)	0.080*	

### Atomic displacement parameters (Å^2^)

**Table:**

	*U*^11^	*U*^22^	*U*^33^	*U*^12^	*U*^13^	*U*^23^
Cl1	0.0551 (3)	0.0522 (3)	0.0583 (3)	0.0181 (2)	−0.0093 (2)	−0.0084 (2)
N1	0.0276 (8)	0.0432 (9)	0.0443 (9)	−0.0003 (6)	−0.0041 (7)	0.0097 (7)
N2	0.0331 (8)	0.0414 (9)	0.0420 (9)	0.0022 (6)	−0.0047 (7)	0.0083 (7)
O1	0.0245 (7)	0.0592 (9)	0.0677 (10)	−0.0014 (6)	−0.0054 (6)	0.0187 (7)
O2	0.0571 (9)	0.0685 (10)	0.0483 (9)	−0.0179 (8)	−0.0210 (7)	0.0156 (7)
O3	0.0616 (10)	0.0567 (9)	0.0430 (8)	0.0006 (7)	−0.0085 (7)	0.0118 (7)
C1	0.0308 (9)	0.0364 (9)	0.0379 (9)	−0.0023 (7)	−0.0092 (7)	−0.0006 (7)
C2	0.0392 (10)	0.0385 (10)	0.0429 (10)	0.0018 (8)	−0.0138 (8)	−0.0023 (8)
C3	0.0627 (14)	0.0394 (11)	0.0541 (13)	0.0000 (10)	−0.0184 (11)	0.0092 (9)
C4	0.0657 (15)	0.0581 (14)	0.0451 (12)	−0.0149 (11)	−0.0081 (11)	0.0158 (10)
C5	0.0485 (12)	0.0648 (14)	0.0419 (11)	−0.0100 (11)	0.0010 (9)	−0.0013 (10)
C6	0.0368 (10)	0.0439 (11)	0.0448 (11)	−0.0008 (8)	−0.0050 (8)	−0.0007 (8)
C7	0.0287 (9)	0.0351 (9)	0.0418 (10)	0.0021 (7)	−0.0035 (7)	0.0007 (7)
C8	0.0342 (10)	0.0437 (11)	0.0451 (11)	0.0025 (8)	−0.0052 (8)	0.0011 (8)
C9	0.0340 (9)	0.0414 (10)	0.0384 (10)	0.0079 (8)	−0.0045 (8)	−0.0012 (8)
C10	0.0365 (10)	0.0459 (11)	0.0361 (10)	0.0039 (8)	−0.0053 (8)	0.0022 (8)
C11	0.0427 (11)	0.0481 (11)	0.0438 (11)	−0.0017 (9)	−0.0044 (9)	0.0039 (9)
C12	0.0431 (11)	0.0450 (11)	0.0357 (10)	0.0107 (8)	−0.0002 (8)	0.0045 (8)
C13	0.0531 (13)	0.0533 (12)	0.0415 (11)	0.0070 (10)	−0.0161 (9)	−0.0002 (9)
C14	0.0446 (11)	0.0469 (11)	0.0473 (11)	0.0012 (9)	−0.0122 (9)	−0.0013 (9)
C15	0.0579 (14)	0.0532 (13)	0.0520 (13)	0.0003 (11)	0.0010 (10)	0.0131 (10)

### Geometric parameters (Å, °)

**Table:**

Cl1—C2	1.741 (2)	C5—C6	1.384 (3)
N1—C7	1.343 (2)	C5—H5	0.9300
N1—N2	1.384 (2)	C6—H6	0.9300
N1—H1	0.897 (10)	C8—C9	1.451 (3)
N2—C8	1.273 (2)	C8—H8	0.9300
O1—C7	1.224 (2)	C9—C14	1.395 (3)
O2—C10	1.352 (2)	C9—C10	1.405 (3)
O2—H2	0.8200	C10—C11	1.388 (3)
O3—C12	1.363 (2)	C11—C12	1.381 (3)
O3—C15	1.426 (3)	C11—H11	0.9300
C1—C2	1.392 (3)	C12—C13	1.390 (3)
C1—C6	1.392 (3)	C13—C14	1.376 (3)
C1—C7	1.495 (2)	C13—H13	0.9300
C2—C3	1.382 (3)	C14—H14	0.9300
C3—C4	1.373 (3)	C15—H15A	0.9600
C3—H3	0.9300	C15—H15B	0.9600
C4—C5	1.376 (3)	C15—H15C	0.9600
C4—H4	0.9300		
			
C7—N1—N2	117.48 (14)	N2—C8—C9	119.94 (18)
C7—N1—H1	123.4 (17)	N2—C8—H8	120.0
N2—N1—H1	119.1 (17)	C9—C8—H8	120.0
C8—N2—N1	118.57 (16)	C14—C9—C10	117.45 (18)
C10—O2—H2	109.5	C14—C9—C8	121.07 (18)
C12—O3—C15	117.32 (17)	C10—C9—C8	121.47 (17)
C2—C1—C6	118.04 (17)	O2—C10—C11	117.14 (17)
C2—C1—C7	122.16 (17)	O2—C10—C9	122.07 (17)
C6—C1—C7	119.72 (16)	C11—C10—C9	120.78 (18)
C3—C2—C1	121.08 (19)	C12—C11—C10	120.03 (19)
C3—C2—Cl1	118.05 (16)	C12—C11—H11	120.0
C1—C2—Cl1	120.83 (15)	C10—C11—H11	120.0
C4—C3—C2	119.7 (2)	O3—C12—C11	123.68 (19)
C4—C3—H3	120.1	O3—C12—C13	115.99 (18)
C2—C3—H3	120.1	C11—C12—C13	120.33 (18)
C3—C4—C5	120.49 (19)	C14—C13—C12	119.22 (18)
C3—C4—H4	119.8	C14—C13—H13	120.4
C5—C4—H4	119.8	C12—C13—H13	120.4
C4—C5—C6	119.8 (2)	C13—C14—C9	122.17 (19)
C4—C5—H5	120.1	C13—C14—H14	118.9
C6—C5—H5	120.1	C9—C14—H14	118.9
C5—C6—C1	120.87 (19)	O3—C15—H15A	109.5
C5—C6—H6	119.6	O3—C15—H15B	109.5
C1—C6—H6	119.6	H15A—C15—H15B	109.5
O1—C7—N1	122.63 (16)	O3—C15—H15C	109.5
O1—C7—C1	122.38 (16)	H15A—C15—H15C	109.5
N1—C7—C1	114.96 (15)	H15B—C15—H15C	109.5

### Hydrogen-bond geometry (Å, °)

**Table:**

*D*—H···*A*	*D*—H	H···*A*	*D*···*A*	*D*—H···*A*
O2—H2···N2	0.82	1.86	2.583 (2)	146
N1—H1···O1^i^	0.90 (1)	1.98 (1)	2.817 (2)	156 (2)

Symmetry codes: (i) *x*−1, *y*, *z*.
